# Pharmacist perceptions of a “good death” and differences in perception between patients with cancer, oncologists, and oncology nurses: a questionnaire survey

**DOI:** 10.1186/s40780-022-00269-4

**Published:** 2023-01-24

**Authors:** Reiko Konishi, Junichi Isogai, Akira Mukai, Koji Komori, Takashi Majima, Shinji Ito, Kou Kawada

**Affiliations:** 1 Department of Pharmacy Practice and Science, Faculty of Pharmaceutical Sciences, Setsunan University, 45-1 Nagaotoge-cho, Hirakata, Osaka, 573-0101 Japan; 2 Department of Hospital Pharmacy, Tsushima City Hospital, 3-73 Tachibana-cho, Tsushima, Aichi 496-8537 Japan; 3 Department of Nursing, Faculty of Allied Health Sciences, Yamato University, 2-5-1 Katayama-cho, Suita, Osaka, 564-0082 Japan

**Keywords:** Good death, Palliative care, Pharmacists, Patients with cancer, Oncologists, Oncology nurses, Views on life and death

## Abstract

**Background:**

For pharmacists expected to encounter the deaths of many of their patients in the near future, it is important to understand the perception of a “good death” for patients with cancer who are likely to be aware of the circumstances of their poor prognosis. In this study, we clarified pharmacists’ perceptions of a “good death” and considered the differences in perception among patients with cancer, oncologists, and oncology nurses.

**Methods:**

From April to June 2022, an anonymous questionnaire survey was conducted on pharmacists working in hospitals and pharmacies and on members of the Japanese Society for Pharmaceutical Palliative Care and Sciences. The questionnaire consisted of 57 questions, called attributes, developed by Miyashita et al. to investigate the perception of “good death” in Japanese cancer medicine. The importance of those attributes was investigated using a 7-point Likert scale.

**Results:**

Three thousand four hundred thirty-two pharmacists were made aware of this survey, and 207 participated in the survey. The responses of pharmacists to the 57 questions were very similar to those of the oncologists. Among them, “Fighting against disease until one’s last moment” and “Not making trouble for others” had very low importance, which was the most significantly different from the responses of patients with cancer. “Fighting against disease until one’s last moment” tended to be significantly underestimated by pharmacists engaged in patient guidance and interview compared to that by pharmacists not engaged in the duty (*p* = 0.02). Also, when we compared pharmacists with or without qualifications related to cancer and palliative care, there was no significant difference in the importance of “Fighting against disease until one’s last moment.” However, the importance of “Not making trouble for others” for qualified pharmacists was significantly underestimated (*p* = 0.04).

**Conclusion:**

Since pharmacists understand the limits of chemotherapy, they may want to be close to the patient but may not strongly agree with the “Fighting against cancer” component that patients with cancer prefer. It may be necessary to reconsider better ways of approaching the wishes and satisfaction of patients with cancer under the care of medical professionals in the field of oncology.

**Supplementary Information:**

The online version contains supplementary material available at 10.1186/s40780-022-00269-4.

## Background

It has been predicted that after 2035, Japan will become a society that will accept many deaths, which is due to a super-aged society. More than ever, many pharmacists and other healthcare providers are expected to encounter the death of a patient. Totoki reports that the attitude and views of life and death of healthcare providers are important when caring for a dying patient [1]. So far, studies on views on life and death have been conducted on doctors, nurses, medical students, and pharmacy students [2–4]. For the measurement, the Rinroshiki scale has been used to measure Japanese views on life and death in a multidimensional and comprehensive evaluation [5]. This scale can evaluate not only the negative aspect of “Death anxiety” but also the positive aspect of “Life purpose.” And it can measure not only attitudes toward death itself, but also attitudes toward death-related events, such as world after death and life span.

Cancer has long been the leading cause of death in Japan. Therefore, the “Cancer Control Act” was enacted, and cancer treatment has been improved and developed [6]. The Cancer Control Act stipulates that necessary measures should be taken to “train specialists” in cancer care, such as physicians and pharmacists, so that standardized cancer treatment can be provided anywhere in Japan. Within that time, the role of pharmacists in cancer treatment has increased, and in recent years, pharmacists have proposed prescriptions for cancer treatment and have even conducted patient interviews prior to physician consultations, as well as provided continued support to patients during home care through telephone follow-up [7, 8]. Meanwhile, our study we conducted revealed that, in assessing pharmacist interviews of outpatients with cancer in the ambulatory chemotherapy unit, there was no significant change in impressions of pharmacists after five interviews. It became clear that the response to adverse events affects impressions and is a factor that should be taken into consideration in building trusting relationships [9]. The pharmacists’ ongoing intervention in patients with cancer provides an environment in which patients can receive cancer treatment with peace of mind. These interventions also extend to palliative care, which is performed until the patient’s death.

It is important for healthcare providers to be able to provide the “end of life” that patients desire or, in other words, a “good death.” Around the year 2000, many studies on the concepts of “quality of death” and “good death” were published for healthcare professionals [10–12]. In 2000, Steinhauser et al. surveyed patients, bereaved families, physicians, and other healthcare professionals in the US about the requirements for a “good death” and found that they included preparation for death, completion of life, contribution to others, and affirmation of the whole person [13, 14]. In 2006, Miyashita et al. surveyed the components of “good death” for patients with cancer, their families, physicians, and nurses in Japan and found a new component, “fighting against cancer,” which was not pointed out in previously reported studies. They further developed 18 components consisting of 57 attributes [15, 16]. Furthermore, Miyashita et al. used a questionnaire composed of those 18 components to survey patients with cancer, oncologists, oncology nurses, and the general population and showed that the “fighting against cancer” desired by patients with cancer is not shared by oncologists and oncology nurses [17].

For pharmacists who are expected to encounter the deaths of many patients in the future, they need to understand the perceptions of a “good death” among patients with cancer who are likely to be aware of the circumstances regarding their death. Furthermore, understanding a “good death” as envisioned by the pharmacist is important for recognizing the differences between their own views and the patient’s. If healthcare providers do not understand the patient’s perception of a “good death,” medical staff may recommend aggressive treatment although the patient desires treatment that emphasizes quality of life, leading to disagreement. Furthermore, the gap in perception between patients and healthcare providers may cause patients to distrust the treatment process and healthcare providers. To support a “good death” in patients with cancer, pharmacists must be included in the multidisciplinary team to provide drug therapy proposals and side effects management. However, pharmacists have recently begun attending to patients’ deathbeds. Thus, there are no surveys or studies comparing perceptions of “good death” among pharmacists and patients with cancer. The purpose of this study was to clarify pharmacists’ perceptions of a “good death” by using the questionnaire that Miyashita et al. used to measure “what kind of death they want to face” [17]. We collected responses from pharmacists using a 57-item questionnaire containing 18 components and compared patterns of pharmacists’ perceptions with responses from patients with cancer, oncologists, and oncology nurses.

## Methods

### Survey procedure

From April to June 2022, via email, we invited pharmacists working at hospitals and community pharmacies near our university and members of the Japanese Society for Pharmaceutical Palliative Care and Sciences to participate in the survey. The requirement for participation was to be involved in support of cancer treatment at least once a week, such as guidance and interviews with patients undergoing cancer treatment. Considering the survey question regarding newly approved anticancer drugs and regimens, we excluded pharmacists who had not provided cancer treatment support such as guidance and interviews for more than 3 years. The URL of the questionnaire created using Google Forms (Google Japan G.K., Tokyo, Japan) was attached via e-mail so that the participants could respond to the survey. A participant could respond anonymously, and the survey described that the responses to the questions were voluntary, that there was no disadvantage if they did not respond, and that they were deemed to have consented to the survey by filling out the questionnaire.

The study was approved by the Setsunan University Ethical Review Committee for Medical and Health Research Involving Human Subjects (Approval Number: 2021-052). In addition, this research was supported by a multicenter study from the Japanese Society for Pharmaceutical Palliative Care and Sciences.

### Participants

As part of the questionnaire, pharmacists were asked to provide their age, gender, years of experience, pharmacy or hospital affiliation, involvement in palliative care, the number of patients with cancer they have treated, the number of experiences of patients with cancer on their deathbed, and their primary involvement in cancer treatment. Methods of involvement included regimen checking, mixing anticancer drugs, guiding and interviewing patients, monitoring treatment efficacy and side effects, prescribing suggestions based on monitoring, and pain management. They were also asked whether they had obtained any certifications related to cancer or palliative care. Qualifications related to cancer treatment included “Board Certified Pharmacist in Oncology Pharmacy,” “Board Certified Oncology Pharmacy Specialist,” “Japanese Society of Pharmaceutical Health Care and Sciences (JSPHCS)-certified Oncology Pharmacist,” “JSPHCS-certified Senior Oncology Pharmacist,” “Accredited Pharmacist of Ambulatory Cancer Chemotherapy,” and “Board-certified Pharmacist of Ambulatory Cancer Chemotherapy.” The qualifications related to palliative care were “Board Certified Pharmacist in Palliative Pharmacy,” “Board Certified Palliative Pharmacy Specialist,” and “Board Certified Provisional Guidance Pharmacist in Palliative Pharmacy.” Pharmacists with either cancer-related or palliative medicine-related qualifications were categorized as “Qualified,” and all others were categorized as “Not Qualified.”

### Measurement of the components of a “good death”

Pharmacists were asked about the importance of the elements that constitute a “good death.” The questionnaire was developed through a qualitative study by Miyashita et al. and consists of 57 questions as attributes that comprise the 18 components of a “good death.” The authors and co-researchers in this study fully agreed on the appropriateness of using this questionnaire in the study.

The 18 components are divided into 10 core components that most Japanese rate as important and 8 optional components that vary in importance based on the individual. That is, the 10 core components are “Physical and psychological comfort,” “Dying in a favorite place,” “Good relationship with medical staff,” “Maintaining hope and pleasure,” “Not being a burden to others,” “Good relationship with family,” “Physical and cognitive control,” “Environmental comfort,” “Being respected as an individual,” and “Life completion.”. The 8 optional components are “Natural death,” “Preparation for death,” “Role accomplishment and contributing to others,” “Unawareness of death,” “Fighting against cancer,” “Pride and beauty,” “Control over the future,” and “Religious and spiritual comfort.”

Examples of the 57 attributes include “Being free from pain and physical distress” and “Being calm,” which constitute the “Physical and psychological comfort” component. For all the 57 attributes, the participants were asked to rate the importance of each item on a 7-point Likert scale (1, absolutely unnecessary; 2, unnecessary; 3, somewhat unnecessary; 4, unsure; 5, somewhat necessary; 6, necessary; and 7, absolutely necessary). They were asked to respond according to their own thoughts and perceptions.

### Statistical analysis

Microsoft Excel 2019 (Microsoft Corp., Redmond, WA, USA) was used for all tabulations and JMP® Pro15 (SAS Institute, Inc., Cary, NC, USA) for statistical analysis. First, pharmacists’ average scores of the 18 components of a “good death” were calculated and compared to those of patients with cancer, oncologists, and oncology nurses reported by Miyashita et al. Next, the proportions of pharmacists who responded with “5, somewhat necessary;” “6, necessary;” and “7, absolutely necessary” for each of the 57 attributes were calculated as “necessary” and compared to those of patients with cancer, oncologists, and oncology nurses. Fisher’s exact test was used to compare the scores of the two groups. Multiple logistic analysis was performed on several items, in particular “Fighting against cancer,” to identify variables associated with pharmacists who rated the item as “necessary.” For example, the choice of “somewhat necessary,” “necessary,” or “absolutely necessary” for “Fighting against disease until one’s last moment,” which is one attribute of “Fighting against cancer,” was used as the objective variable, and variables related to the attributes of pharmacists were used as explanatory variables. A significance level of 0.05 was set, and two-tailed tests were performed for all analyses.

The data of patients with cancer, oncologists, and oncology nurses used for comparison were those reported by Miyashita et al. In their report, the survey was conducted from February to April 2008 on patients with cancer who visited the outpatient department of the radiation oncology department at the National University Hospital, as well as oncologists and oncology nurses working at the hospital.

## Results

### Participant characteristics

Through direct contact and a mailing list requesting participation, 3,432 pharmacists were made aware of this survey, and 207 participated in the survey. The characteristics of the participants are shown in Table 1. There was no gender bias in the number of participants, and the largest age distribution was 40–49 with 37.2%, and 92.2% of participants were between the ages of 30 and 59. The proportion of the participants who reported that they had more than 10 years of clinical experience was 83.1%, of which 34.8%, the highest percentage, reported that they had more than 20 years of clinical experience. The proportion of the participants who reported being involved in palliative care was 79.2%, and those who reported working in hospitals were 67.6%. The proportion of the participants who had been involved in the treatment of more than 100 patients with cancer was 70.1%, and those who reported more than 200 patients were 46.9%. While 15.5% of participants had no experience of attending to the deathbed of a patient with cancer, 53.6% of participants had experienced that for 20 or more patients with cancer.

**Table 1:** Participants’ attributes

	*n* = 207	%
Gender		
Male	96	46.4
Age		
20–29	11	5.3
30–39	74	35.7
40–49	77	37.2
50–59	40	19.3
60–69	5	2.4
Clinical experience		
≤ 2	6	2.9
3–4	6	2.9
5–9	23	11.1
10–14	51	24.6
15–19	49	23.7
≥20	72	34.8
Affiliated		
Hospital		
Not involved in palliative care	34	16.4
Involved in palliative care	140	67.6
Pharmacy		
Not involved in palliative care	9	4.3
Involved in palliative care	24	11.6
Experience in treatment of patients with cancer^a^		
0	5	2.4
1–9	11	5.3
10–19	10	4.8
20–49	17	8.2
50–99	19	9.2
100–199	48	23.2
≥200	97	46.9
Experience of attending to the deathbed of patients with cancer		
0	32	15.5
1–9	46	22.2
10–19	18	8.7
20–49	30	14.5
50–99	35	16.9
100–199	21	10.1
≥200	25	12.1

^a^ “Treatment” refers to explanation, guidance, and interview

### Average of importance in the “good death” component

The means and standard deviations of the 18 components of a “good death” were calculated, and the differences among pharmacists and patients with cancer, oncologists, and oncology nurses are shown in Table 2. Of the 18 components, “Fighting against cancer” had the largest difference compared with patients with cancer, with a mean difference of 1.9. The next item with a large difference was “Not being a burden to others,” with a mean difference of 1.3. On the other hand, pharmacists’ responses to “Life completion,” “Preparation for death,” and “Control over the future” showed smaller differences compared with those of patients with cancer. Among patients with cancer, oncologists, and oncology nurses, the oncologists’ scores of the 18 components were similar. The largest difference with oncologists was “Not being a burden to others,” with a mean difference of 0.7. Almost all of the oncology nurses’ scores of the 18 components were higher than the pharmacists’ scores, with the largest difference observed for “Dying in a favorite place” and “Good relationship with family” (mean difference, 0.6).

**Table 2:**
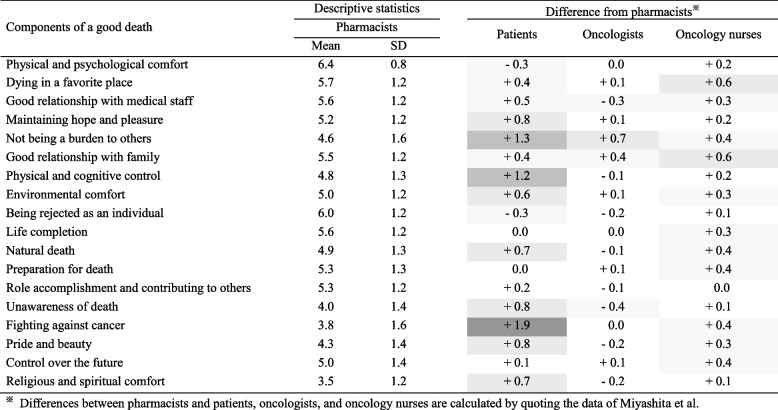
Comparison of the importance of good death components

### Proportion of participants who responded “necessary” for each attribute of “good death”

The proportions of participants who responded “necessary” in all of the attributes comprising “good death” were calculated, and differences between patients with cancer, oncologists, and oncology nurses are shown (See Additional file 1). Table 3 shows only those attributes that differed by a factor of 2 or more between patients with cancer and pharmacists. When comparing pharmacists to patients with cancer, oncologists, and oncology nurses, the overall proportions were similar to those of oncologists. All of the attributes presented in the table were rated lower by pharmacists than by patients. In particular, the majority of patients with cancer responded that “Fighting against disease until one’s last moment” was necessary, while only 22% of pharmacists responded that it was necessary. Similarly, for “Not making trouble for others” and “Living as long as possible,” the proportion of pharmacists who answered “necessary” differed from that of patients with cancer. The proportion of pharmacists in each attribute was 39% and 19%, respectively, and that of patients with cancer was 48% and 44% higher than pharmacists, respectively.

**Table 3:** Comparison of attributes of a good death

Components of a good death	Attributes of a good death	Proportion (%)	Difference from pharmacists (%)^a^		
		Pharmacists	Patients	Oncologists	Oncology nurses
Fighting against cancer	Fighting against disease until one’s last moment	22	+ 59	− 3	+ 8
Not being a burden to others	Not making trouble for others	39	+ 48	+ 29	+ 17
Fighting against cancer	Living as long as possible	19	+ 44	0	+ 5
Pride and beauty	Not exposing one’s physical and mental weakness to anyone else	22	+ 44	+ 4	+ 8
Religious and spiritual comfort	Feeling that one is protected by higher power beyond oneself	19	+ 30	+ 1	+ 8
Unawareness of death	Dying without awareness that one is dying	20	+ 29	+ 1	+ 4
Unawareness of death	Not being informed of bad news	18	+ 22	− 5	+ 4
Religious and spiritual comfort	Having faith	11	+ 20	+ 8	+ 6

Percentage of respondents who answered somewhat necessary, 5; necessary, 6; and absolutely necessary, 7 on a 7-point scale

^a^ Differences between pharmacists and patients, oncologists, and oncology nurses are calculated by quoting the data of Miyashita et al

In particular, pharmacists’ results on “Not making trouble for others” differed from those of the oncologists or oncology nurses. Pharmacists had the lowest proportion of “Not making trouble for others” at 39%, and that of oncologists and oncology nurses was 29% and 17% higher, respectively. All proportions for oncology nurses were 4–8% higher than that of pharmacists, except for “Not making trouble for others.”

### Pharmacists’ duties affect the necessity of “Fighting against disease until one’s last moment”

The proportions of pharmacists who indicated that “Fighting against disease until one’s last moment,” the attribute with the largest difference from the proportion of patients with cancer, is shown in Table 4 for each duty in which the participants were engaged in. “Regimen registration” was the least reported duty the respondents were engaged in. Among seven duties, approximately 20% of pharmacists who answered “engaged” rated “Fighting against disease until one’s last moment” as high. Furthermore, pharmacists who answered “not engaged” responded that they were “necessary” at a higher proportion than pharmacists who answered engaged across all duties. In particular, 38% of pharmacists who answered not engaged for “Patient guidance and interview,” responded “necessary,” which was significantly higher than 19% who engaged in it (*p* = 0.02).

**Table 4:** Respondents who answered “necessary” for “Fighting against disease until one’s last moment” by pharmacists’ duties

Pharmacist duties	n	number (%)	*P*-value
Regimen registration			
Engaged in	65	13 (20)	0.72
Not engaged	142	33 (23)	
Check dosage and interval between doses of anticancer drugs			
Engaged in	122	25 (20)	0.50
Not engaged	85	21 (25)	
Mixing of anticancer drugs			
Engaged in	91	19 (21)	0.74
Not engaged	116	27 (23)	
Patient guidance and interview			
Engaged in	170	32 (19)	0.02
Not engaged	37	14 (38)	
Monitoring of treatment efficacy and side effects			
Engaged in	161	33 (20)	0.31
Not engaged	46	13 (28)	
Prescribing suggestions based on monitoring			
Engaged in	138	28 (20)	0.38
Not engaged	69	18 (26)	
Pain management			
Engaged in	176	36 (20)	0.14
Not engaged	31	10 (32)	

Significance test was performed by Fisher’s exact test

Table 5 shows the results of a multiple regression analysis evaluating pharmacist duty as a factor in the proportion of respondents who indicated that “Fighting against disease until one’s last moment” is necessary. “Patient guidance and interview” were observed as a significant factor with an odds ratio of 0.31 (*p* = 0.02). Although no significant differences were observed, the odds ratios for “Monitoring of treatment effects and side effects” and “Prescribing suggestions based on monitoring” were 1.29 and 1.34, respectively, while the odds ratio for “Pain management” was 0.59.

**Table 5:** “Necessary” responses for “Fighting against disease until one’s last moment” by pharmacist duties

Pharmacist duties	OR	95% CI	*P*-value
Regimen registration	1.00	0.41–2.43	0.99
Check dosage and interval between doses of anticancer drugs	0.92	0.41–2.06	0.84
Mixing of anticancer drugs	0.91	0.41–1.99	0.81
Patient guidance and interview	0.31	0.10–0.91	0.03
Monitoring of treatment efficacy and side effects	1.29	0.34–4.95	0.71
Prescribing suggestions based on monitoring	1.34	0.40–4.48	0.64
Pain management	0.59	0.23–1.49	0.27

Significance test was performed by multiple regression test

*OR* Odds ratio, *CI* Confidence interval

### Influence of cancer and palliative care-related qualifications on the necessity of “Fighting against disease until one’s last moment” and “Not making trouble for others”

Attributes that were very different from that of the patients were “Not making trouble for others” and “Fighting against disease until one’s last moment.” Table 6 shows the proportion of pharmacists with and without cancer or palliative care-related qualifications who responded “necessary” for “Fighting against disease until one’s last moment” or “Not making trouble for others.” However, these results were analyzed, excluding the three respondents who did not respond to the question about qualifications. Pharmacists with either cancer-related or palliative care-related qualifications were considered “Qualified.” Pharmacists with these qualifications were compared to those without them. In addition, pharmacists with and without cancer-related and palliative care-related qualifications were compared for each component score. For “Fighting against disease until one’s last moment” and “Not making trouble for others,” 25% and 46% of pharmacists without qualifications, respectively, answered necessary, which was higher than that of pharmacists with qualifications. No significant differences were observed in the proportion of respondents who responded “Fighting against disease until one’s last moment” as “necessary” by qualifications. On the other hand, in the “Not making trouble for others” attribute, 32% of pharmacists with qualifications answered necessary, which was significantly lower than that of pharmacists without qualifications (*p* = 0.04).

**Table 6:** Number of respondents who gave a score of 5 or more for qualifications

	n	Fighting against disease until one’s last moment		Not making trouble for others	
		number (%)	*P*-value	number (%)	*P*-value
Qualifications					
With	109	21 (19)	0.32	35 (32)	0.04
Without	95	24 (25)		44 (46)	
Qualifications related to cancer					
With	64	11 (17)	0.28	21 (33)	0.28
Without	140	34 (24)		58 (41)	
Qualifications related to palliative care					
With	80	16 (20)	0.61	26 (33)	0.19
Without	124	29 (23)		53 (43)	

Excluded 3 subjects who did not answer. Significance test was performed by Fisher’s exact test

Qualifications related to cancer: Board Certified Pharmacist in Oncology Pharmacy, Board Certified Oncology Pharmacy Specialist, JSPHCS-certified Oncology Pharmacist, JSPHCS-certified Senior Oncology Pharmacist, Accredited Pharmacist of Ambulatory Cancer Chemotherapy, Board-certified Pharmacist of Ambulatory Cancer Chemotherapy

Qualifications related to palliative care: Board Certified Pharmacist in Palliative Pharmacy, Board Certified Palliative Pharmacy Specialist, Board Certified Provisional Guidance Pharmacist in Palliative Pharmacy

## Discussion

Pharmacists involved in cancer treatment need to understand the patient’s perception of death and discuss it with the patient and family to support each individual’s quality of life. In this study, we asked pharmacists for the first time about their perceptions of the components of a “good death.” The results provide insight into how pharmacists should approach dying patients.

In 2000, Steinhauser et al. surveyed US patients on the components that make up a “good death” and found strong support for the importance of spirituality, including “Not being a burden to others” and “Being able to help others” [13]. In addition, Miyashita et al. identified “Fighting against cancer” as a component of a “good death” in Japanese patients, which was not found in the previous studies [15, 16]. Decades ago, medical paternalism was the norm in Japan, where medical decisions were left to physicians [16]. As a result, elderly patients in Japan tended to be very afraid of being abandoned by their physicians [18, 19]. Against this background, it has been reported that patients are favorable toward “Fighting against cancer” and are especially eager toward “Fighting against disease until one’s last moment” [17].

In this study, among the 18 components of a “good death,” “Life completion,” “Preparation for death,” and “Control over the future” were perceived similarly by pharmacists and patients with cancer, although all other components differed, and the greatest difference was found in “Fighting against cancer.” In addition, pharmacists’ scores of the 18 components were closer to those of oncologists than those of oncology nurses, suggesting that their perceptions are similar to those of oncologists. As a multimodal therapy in cancer treatment, chemotherapy may be performed along with surgery and radiation therapy with the expectation of cure or may be performed for the purpose of prolonging life or alleviating symptoms. Pharmacists play a major assistive role during chemotherapy for patients undergoing cancer treatment. Most oncology nurses know that chemotherapy has a negative impact on a patient’s quality of life [20]. Oncologists know the effectiveness and limits of surgery and radiation therapy as well as chemotherapy. Like oncologists, pharmacists understand the limits of chemotherapy and its side effects, based on guidelines. The results of this analysis showed that the number of years of experience did not affect the perception of a “good death” (data not shown). A previous study reported that understanding of chemotherapy is not related to years of clinical experience [21]. On the other hand, the distribution of the number of years of clinical experience was similar between the oncologists included in the study by Miyashita et al. and the pharmacists included in our study [17]. These background characteristics, including of knowledge and clinical experience, may have differentiated the pharmacists’ results from the patients’ results and brought them closer to the oncologists’ results.

According to Miyashita et al.’s report, the components of “Fighting against cancer” consist of three attributes: “Fighting against disease until one’s last moment,” “Believing that one used all available treatments,” and “Living as long as possible” [15, 16]. Of these, the most significant difference in patients was “Fighting the disease until one’s last moment.” Using this attribute as an indicator to analyze the influence of the pharmacists’ duties yielded interesting information. Across all duties, more pharmacists who responded “not engaged” rated “Fighting against disease until one’s last moment” as necessary than that by pharmacists who responded “engaged.” The results suggest that pharmacists who are not engaged in these duties are closer to the patients’ perceptions, and it is possible that pharmacists who are engaged in duties related to cancer treatment may understand the efficacy and limitations and diverge from the patients’ perceptions. In particular, the odds ratio of pharmacists engaged in “Patient guidance and interview” and “Pain management,” which focus on understanding the pain and feelings of the patient, was less than 1. These pharmacists may not have considered “Fighting against disease until one’s last moment” as necessary due to engagement in work, which prevents their understanding of the pain and feelings of patients. Pharmacists engaged in “Monitoring of treatment efficacy and side effects” and “Pain management” may focus more on the data and evidence rather than the patient’s complaints in support of aggressive treatment. Although no significant differences were observed, pharmacists engaged in “Monitoring of treatment effects and side effects” and “Prescribing suggestions based on monitoring” were more likely to favor “Fighting against disease until one’s last moment.”

“Not being a burden to others” is another component of this study that deserves special mention. It corresponds to the attribute “Not making trouble for others” and was perceived by oncologists and oncology nurses similarly to patients with cancer. Only pharmacists had different perceptions, and a low proportion of pharmacists rated this as necessary. Palliative care, including cancer treatment, cannot be completed by a single healthcare provider [22]. It has also been reported that patients with cancer are willing to undergo aggressive treatment, including chemotherapy, until the last moment of their life, even if the possibility of cure is low [20, 23, 24]. When considering communication with such patients who are likely to be aware of death as a possibility, oncologists are at risk of disappointing patients by discontinuing the administration of anticancer drugs [18, 19]. In addition, nurses appear to be stressed out approaching patients in these situations [25]. In Japan, pharmacists, on the other hand, have only been stationed in hospital wards in recent years and may have accepted that patients usually rely on health care providers. This difference in communication with patients with cancer may have reduced the proportion of pharmacists’ perceiving “Not making trouble for others” as a necessary component of a “good death.” This can be inferred from the fact that this was more pronounced among pharmacists with qualifications related to cancer or palliative care. Qualified pharmacists may be more likely to want to contribute to patients.

The results of this study promote understanding regarding the differences in perceptions of a “good death” between pharmacists and patients with cancer, will lead to reconsideration of the diversity of ways that patients with cancer choose to spend their days until death, and indicate that diversity of interventions by pharmacists may be required. We believe that pharmacists’ perceptions do not have to match those of patients with cancer or of other healthcare providers. Understanding that the perceptions of oncologists, oncology nurses, and pharmacists do not always align will enable clinicians to treat patients with cancer with diverse ways of thinking according to their occupational ability. Once again, we should understand each patient’s desire for cancer treatment.

This study has several limitations. Duties related to cancer treatment differed between hospitals and pharmacies, with fewer respondents belonging to pharmacies. Furthermore, the response rate was low at approximately 6%; only highly motivated pharmacists may have responded. Additionally, the data of patients with cancer, oncologists, and oncology nurses that were compared in this study were obtained in 2008, and the participants were recruited from only one university. The types of cancer among patients with cancer included head and neck (26%) and uterine (16%) cancer, and the majority of patients received radical radiation therapy; 19% had metastasis and most patients received follow-up care. Since 2008, the medical environment surrounding patients with cancer has changed significantly, and it is possible that patients with cancer perceptions of a “good death” has also changed. Thus, the results of this study may be considered uncommon due to a limited population and may not be applied to the general population. Additionally, the “last” in the attribute “Fighting against disease until one’s last moment” could have been perceived differently by patients with cancer and pharmacists, i.e., it is possible that a patient with cancer may think that the fight will continue beyond treatment to the very last moment, whereas the pharmacist may only think until the treatment period has ended. This ambiguity in wording may have skewed the responses of participants.

## Conclusion

For pharmacists, who now have more contact with patients and will attend to more patient deathbeds in the future, more than ever before, understanding the patient’s view of life and death is necessary for patients to be able to rely on them. When we compared groups with many patients undergoing follow-up, there was a significant difference between pharmacists and the patients regarding the recognition of “Fighting against disease until one’s last moment” and “Not making trouble for others.” It is important for pharmacists to understand the potential gaps in perceptions of a “good death” when caring for patients with cancer in any population. In order to comply with the patient’s desires and for patient satisfaction, an educational approach may be required, such as providing opportunities for patients with cancer and their families to share their lifestyles and thoughts by participating in cancer association meetings during the qualification process. Further study is needed.

## Supplementary Information

**Additional file 1.** Difference in attributes answered “necessary” between pharmacists and patients with cancer, oncologists, and oncology nurses.

## Data Availability

All data generated or analyzed during this study are included in this published article and its supplementary information files.

## Abbreviation

JSPHCS: Japanese Society of Pharmaceutical Health Care and Sciences
