# Supplementary material for: Pharmacist perceptions of a “good death” and differences in perception between patients with cancer, oncologists, and oncology nurses: a questionnaire survey
Source: J Pharm Health Care Sci. 2023 Jan 24;9:5. doi: 10.1186/s40780-022-00269-4 (PMC9875416; doi:10.1186/s40780-022-00269-4)
Supplement: Supplementary file 1 — Additional file 1. Difference in attributes answered “necessary” between pharmacists and patients with cancer, oncologists, and oncology nurses. [file 40780_2022_269_MOESM1_ESM.docx]

Additional Table 1　Difference in attributes answered “necessary” between pharmacists and patients, oncologists, and oncology nurses

| Components | Attributes | Proportion (%) |  | Difference from pharmacists (%)^a^ | | |
| --- | --- | --- | --- | --- | --- | --- |
|  |  | Pharmacists |  | Patients | Oncologists | Oncology nurses |
| Physical and psychological comfort | Being free from pain and physical distress | 98 |  | - 6 | + 2 | + 1 |
|  | Being calm | 98 |  | - 1 | 0 | + 1 |
| Dying in a favorite place | Being able to stay at one’s favorite place | 88 |  | + 5 | + 5 | + 7 |
| Good relationship with medical staff | Trusting physician | 86 |  | + 11 | + 4 | + 9 |
|  | Discussing one’s treatment with one’s physician | 95 |  | + 3 | - 6 | + 2 |
|  | Having people who listen to me | 93 |  | + 2 | + 2 | + 5 |
|  | Receiving consistent care from the same physician and nurse | 58 |  | + 33 | - 10 | + 15 |
|  | Having a physician or nurse with whom one can discuss fears of death | 89 |  | - 17 | - 30 | - 5 |
|  | Having a nurse with whom one feels comfortable | 89 |  | + 7 | - 4 | + 2 |
| Maintaining hope and pleasure | Living positively | 65 |  | + 30 | + 10 | + 9 |
|  | Having some pleasure in daily life | 86 |  | + 10 | - 5 | + 2 |
|  | Living in hope | 73 |  | + 20 | - 5 | + 1 |
| Not being a burden to others | Not making trouble for others | 39 |  | + 48 | + 29 | + 17 |
|  | Not being a burden to family members | 53 |  | + 35 | + 20 | + 14 |
|  | Having no financial worries | 74 |  | + 21 | + 10 | + 6 |
| Good relationship with family | Having family support | 79 |  | + 14 | + 8 | + 15 |
|  | Having family to whom one can express one’s feeling | 88 |  | + 5 | + 3 | + 6 |
|  | Spending enough time with one’s family | 77 |  | + 12 | + 13 | + 16 |
|  | Family is prepared for one’s death | 88 |  | + 2 | + 1 | + 6 |
|  | Believing that one’s family will do well after one’s death | 87 |  | + 4 | + 8 | + 6 |
|  | Having family by one’s side when one is going to die | 79 |  | + 6 | + 3 | + 13 |
| Physical and cognitive control | Being independent in daily activities | 56 |  | + 37 | - 2 | + 9 |
|  | Being able to eat | 64 |  | + 31 | - 5 | + 1 |
|  | Being mentally clear | 66 |  | + 32 | + 6 | + 6 |
| Environmental comfort | Living like being at home | 66 |  | + 21 | - 1 | + 7 |
|  | Living in calm circumstances | 64 |  | + 21 | + 13 | + 18 |
| Being respected as an individual | Not being treated as an object or a child | 95 |  | - 8 | + 2 | + 3 |
|  | Being free from trivial routines | 77 |  | + 4 | - 5 | + 8 |
|  | Being respected for one’s values | 96 |  | - 7 | - 1 | 0 |
| Life completion | Feeling that one’s life was completed | 83 |  | - 7 | - 3 | + 6 |
|  | Family has no regrets for one’s death | 84 |  | + 2 | - 6 | + 8 |
|  | Having no regrets | 83 |  | - 2 | - 4 | + 4 |
| Natural death | Not being connected to medical instruments or tubes | 51 |  | + 18 | + 4 | + 12 |
|  | Dying a natural death | 72 |  | + 16 | - 7 | + 9 |
| Preparation for death | Being prepared for dying | 87 |  | - 4 | 0 | + 5 |
|  | Seeing people whom one wants to see | 88 |  | - 18 | - 3 | + 4 |
|  | Saying good-bye to dear people | 84 |  | - 13 | - 1 | + 7 |
|  | Feeling thankful to people | 80 |  | + 14 | + 13 | + 11 |
|  | Being reconciled with people | 41 |  | + 12 | - 3 | + 14 |
| Role accomplishment and contributing to others | Feeling that one can contribute to others | 68 |  | + 2 | - 6 | - 4 |
|  | Maintaining one’s role in family or occupational circumstances | 78 |  | + 5 | - 3 | - 3 |
|  | Feeling that one’s life is worth living | 87 |  | + 2 | - 7 | - 2 |
| Unawareness of death | Not being informed of bad news | 18 |  | + 22 | - 5 | + 4 |
|  | Dying without awareness that one is dying | 20 |  | + 29 | + 1 | + 4 |
|  | Living as usual without thinking about death | 62 |  | + 26 | - 18 | - 4 |
| Fighting against cancer | Fighting against disease until one’s last moment | 22 |  | + 59 | - 3 | + 8 |
|  | Believing that one used all available treatments | 55 |  | + 37 | - 4 | + 2 |
|  | Living as long as possible | 19 |  | + 44 | 0 | + 5 |
| Pride and beauty | Not receiving pity from others | 69 |  | + 3 | - 20 | - 5 |
|  | Not having a change in one’s appearance | 45 |  | + 25 | - 16 | + 16 |
|  | Not exposing one’s physical and mental weakness to anyone else | 22 |  | + 44 | + 4 | + 8 |
| Control over the future | Controlling time of death, like euthanasia | 49 |  | - 12 | - 4 | - 19 |
|  | Having the planned arrangements of one’s grave, funeral, and last will | 67 |  | + 5 | + 6 | 0 |
|  | Knowing how long one will live | 76 |  | - 7 | + 13 | + 9 |
|  | Knowing what to expect about one’s condition in the future | 75 |  | - 12 | + 4 | + 4 |
| Religious and spiritual comfort | Having faith | 11 |  | + 20 | + 8 | + 6 |
|  | Feeling that one is protected by higher power beyond oneself | 19 |  | + 30 | + 1 | + 8 |
| ^a^ Differences between pharmacists and patients, oncologists, and oncology nurses were calculated according to the method described by Miyashita et al [17].  Percentage of respondents who answered, somewhat necessary, 5; necessary, 6; and absolutely necessary, 7 on a 7-point scale. | | | | | | |
